# Antibiotic Sensitivity and Plasmid Profiles of *Escherichia coli* Isolated from Pediatric Diarrhea

**DOI:** 10.4103/0974-777X.56255

**Published:** 2009

**Authors:** Babu Uma, Kesani Prabhakar, Saddayappan Rajendran, Kannaiyan Kavitha, Yelavarthi Lakshmi Sarayu

**Affiliations:** *Division of Microbiology, Rajah Muthiah Medical College and Hospital, Annamalainagar, India*

**Keywords:** Antibiotic sensitivity, Diarrhea, *E. coli*, Plasmids, Transconjugants

## Abstract

**Background:**

The emergence of drug resistance among diarrheagenic *Escherichia coli (E. coli)* in the pediatric population is an important cause of morbidity and mortality in developing countries.

**Material and Methods:**

Isolation and identification of *E. coli* strains from stool specimens are carried out according to standard techniques. Antibiotic susceptibility testing was performed using disc-diffusion method. Plasmid profiling and conjugation experiments were done to analyze the antibiotic resistance transfer from one bacterium cell to another through plasmid.

**Results:**

Out of 170 pediatric diarrheal samples, 105 (61.76%) *E. coli* strains were isolated. About 90% of *E. coli* strains were resistant to most of the antimicrobial agents tested. All the isolates were resistant to ampicillin, imipenem and cotrimoxazole and were sensitive to amikacin. The resistance to antibiotics shows 29 different antibiotic resistance patterns. About 67 (64%) strains of *E. coli* isolates harbored plasmids, and 51 (76.1%) of them were able to transfer their plasmids. The plasmid sizes ranged from 1.0 to 25 kb, the most common plasmid of size 4.8 kb being detected in all the plasmid-harbored *E. coli* strains. The results of transconjugation show that all the transconjugant colonies were carrying 4.8-kb plasmid and were resistant to ampicillin, imipenem and cotrimoxazole.

**Conclusion:**

There is an increase in the prevalence of drug resistance among *E. coli* isolates, and conjugal transfer of plasmids has greatly contributed to the rapid spread of antibiotic resistance among *E. coli* isolates.

## INTRODUCTION

Diarrhea is an important cause of morbidity and mortality in the pediatric population of developing countries.[1] In India, diarrheal infections are most commonly caused by *Escherichia coli (E. coli)*, with occasional outbreaks.[2] *E. coli* strains have been associated with a number of disease syndromes; among these, often severe and fatal infections include pyelonephritis, septicemia, meningitis, endocarditis, urinary tract infections and epidemic diarrhea in adults and children. The major biotypes of *E. coli* in diarrhea are enterotoxigenic *E. coli* (ETEC), enteropathogenic *E. coli* (EPEC), enterohemorrhagic *E. coli* (EHEC), enteroinvasive *E. coli* (EIEC), enteroaggregative *E. coli* (EAEC), and enteroadherent *E. coli* or diffusely adhering *E. coli* (DAEC).[3]

Fecal, oral and food-borne transmissions of *E. coli* have been well documented.[4] Bacterial resistance to commonly used antibiotics is a threat to public health throughout the world. Multiple antibiotic resistances in bacteria are most commonly associated with the presence of plasmids which contain one or more resistance genes. Transmission of resistance genes from normally more virulent pathogenic species to nonpathogenic organisms is very common with the animal and human intestinal tract micro flora.[5] Furthermore, the use of antibiotics perpetuated antibiotic resistant plasmids in countries like India, where there is an unrestricted use of antibiotics. In this work, we evaluated the resistance profile of *E. coli* isolates to commonly used antibiotics and performed plasmid profiles.

## MATERIAL AND METHODS

The feces samples were collected from 170 patients below 5 years of age with diarrhea attending the Rajah Muthiah Medical College and Hospital, Annamalainagar, Tamil Nadu, India. Standard procedures were followed for isolation and confirmation of E. *coli* strains using various biochemical reactions.[6] Serotyping was done at the Central Research Institute, Kasuali, Himachal Pradesh, India.

### Antibiotic susceptibility testing

Susceptibility of isolated *E. coli* strains to different antibiotics was determined by Kirby-Bauer disc-diffusion technique[7] as specified by the National Committee for Clinical Laboratory Standards (NCCLS).[8] The following antibiotics were used: ampicillin (10 μg), amikacin (30 μg), chloramphenicol (30 μg), ciprofloxacin (5 μg), norfloxacin (10 μg), nalidixic acid (30 μg), cotrimoxazole (10 μg), imipenem (10 μg), cefotaxime (30 μg), ceftriaxone (30 μg) and ceftazidime (30 μg). *E. coli* ATCC 25922 was used as a control.

### Conjugation studies

Conjugational transfer was carried out using drug-resistant *E. coli* as donor and *E. coli* k12 plasmid-free strain resistant to kanamycin as the recipient, as described by Shohayeb *et al.*[9] The donors and recipient *E. coli* cells were grown to logarithmic phase, mixed together in nutrient broth. Conjugation was allowed to take place for 48 hours at 37°C. The mating cells were subcultured into nutrient agar plates containing ampicillin (50 μg/mL) and kanamycin (50 μg/mL) to inhibit the growth of the donor and the recipient. Resistant character was determined by testing all transformants against all antibiotics to which donor strains were resistant. Conjugation was confirmed as positive only when resistant transconjugants were shown to contain a plasmid of a size similar to that found in the original isolate.

### Isolation and separation of plasmid DNA

Plasmid DNA was extracted from both donor and transconjugants. Small-scale alkaline lysis method was used as described by Sambrook *et al.*[10] Extracted plasmids were electrophoresed for 2 hours in a horizontal 0.8% agarose gel with pH 8.0 TBE buffers.[11] The gels were stained with ethidium bromide 0.5 μg/mL for 20 minutes, and bands were visualized by UV transilluminator. Lambda DNA digested with Hind 111 and EcoRI was used as the DNA standard marker.

## RESULTS AND DISCUSSION

Out of 170 pediatric diarrheal samples, 105 (61.76%) *E. coli* strains were isolated. Based on unpublished data, the prevalence of cases of diarrhea in Chidambaram, Tamil Nadu, with a known etiology was 71.83% in summer and 63.1% in monsoon. Diarrhea in children in developing countries has been reported in 50% to 60% of diagnosed cases.[12,13] The proportion of diarrheagenic *E. coli* in Chidambaram was high as compared with that in previous reports from developing countries, as well as from India.[14,15] In recent years, it has become clear that *E. coli* play an important role in the etiology of acute diarrhea.[16,17] The major serogroups identified were as follows: 26 strains (24.8%) were O12 serogroup, 21 strains (20%) were O25 serogroup, and 20 (19%) strains were belonged to O60 serogroup. About 10 (9.5%) strains of *E. coli* were untypable. The resistance to antibiotics shows 29 different antibiotic resistance patterns. Among them, 18 isolates were resistant to ampicillin, imipenem and cotrimoxazole alone.

The data presented in Table 1 highlights the emergence of a high rate of drug resistance in *E. coli* to the common antimicrobial agents used in the treatment of diarrhea. All the isolates were resistant to ampicillin, imipenem and cotrimoxazole and were sensitive to amikacin. The prevalence of drug resistance to ampicillin, chloramphenicol, cotrimoxazole, imipenem, nalidixic acid, norfloxacin was “very high” in our study. This may be due to the indiscriminate use of first-line inexpensive antibiotics in our country. The resistance to ampicillin and imipenem may be due to production of beta-lactamases enzymes, and the most common mechanism for resistance to cotrimoxazole is acquisition of plasmid-encoded, variant diamino-pyrimidine folate reductase enzymes.[18] This may be chromosomal or plasmid mediated. Recently resistances to third-generation cephalosporins have emerged as a major concern, as seen in this study. In India, the emergence of multidrug resistant strains and its variation over the years have been increasing.[14] Appropriate antibiotic therapy for diarrhea reduces mortality and also shortens the duration of symptoms. Increased frequency of drug-resistant *E. coli* strains is remarkable, since resistance to first-line drugs will require more expensive drugs for effective treatment and may pose a major challenge to the health care system.

**Table 1:** Antibiotic-resistance patterns of *E. coli* isolates and plasmid analysis

Antimicrobial resistance pattern	No. of isolates (%)	No. of strains with plasmids
Ap, I, Co	18 (17.14)	11
Ap, I, Co, Chl	5 (4.76)	5
Ap, I, Co, Na	2 (1.9)	2
Ap, I, Co, Nor	9 (8.57)	5
Ap, I, Co, Cip	5 (4.76)	3
Ap, I, Co, Chl, Na	2 (1.9)	2
Ap, I, Co, Chl, Cip	4 (3.81)	3
Ap, I, Co, Na, Ca	5 (4.76)	3
Ap, I, Co, Cip, Nor	6 (5.71)	1
Ap, I, Co, Cip, Ca	8 (7.62)	4
Ap, I, Co, Na, Nor	1 (0.95)	1
Ap, I, Co, Ca, Ci	1 (0.95)	1
Ap, I, Co, Na, Ci	1 (0.95)	1
Ap, I, Co, Ca, Ce	2 (1.9)	1
Ap, I, Co, Chl, Na, Nor	2 (1.9)	2
Ap, I, Co, Chl, Cip, Ca	1 (0.95)	-
Ap, I, Co, Chl, Cip, Ce	4 (3.81)	3
Ap, I, Co, Chl, Nor, Ce	2 (1.9)	2
Ap, I, Co, Na, Cip, Ce	3 (2.86)	2
Ap, I, Co, Na, Cip, Ca	2 (1.9)	1
Ap, I, Co, Na, Ca, Ci	3 (2.86)	2
Ap, I, Co, Na, Cip, Ci	2 (1.9)	2
Ap, I, Co, Na, Nor, Ca	2 (1.9)	2
Ap, I, Co, Na, Nor, Cip	2 (1.9)	1
Ap, I, Co, Na, Ce, Ca	1 (0.95)	1
Ap, I, Co, Nor, Cip, Ca	3 (2.86)	2
Ap, I, Co, Cip, Ca, Ca	2 (1.9)	-
Ap, I, Co, Cip, Ci, Ce	2 (1.9)	1
Ap, I, Co, Chl, Na, Cip, Ca, Ci	5 (4.76)	3

Ap: Ampicillin, I: Imipenem, Co: Cotrimoxazole, Chl: Chloramphenicol, Cip: Ciprofloxacin, Na: Nalidixic acid, Nor: Norfloxacin, Ca: Cefotaxime, Ce: Ceftazidime, Ci: Ceftriaxone

Approximately 67 (64%) *E. coli* isolates harbored plasmids, and 51 (76.1%) of them were able to transfer their plasmids. The plasmid size ranged from 1.0 to 25 kb, the most common plasmid of size 4.8 kb being detected in all the plasmid-harbored *E. coli* strains. Plasmid profile analysis has been widely used in epidemiological investigations.[19] Holmberg *et al.*[20] reported that plasmid analysis was at least as specific as phage typing. The resistances to antibiotics other than ampicillin, imipenem and cotrimoxazole among *E. coli* strains were compared with those with and without plasmids, and it was observed that there is high frequency of resistance among *E. coli* strains with plasmids than without plasmids. There appears to be a constant relationship between a particular plasmid and resistance to antimicrobial agents tested, indicating that there is a potential spread of resistance by conjugation.

**Figure 1:**
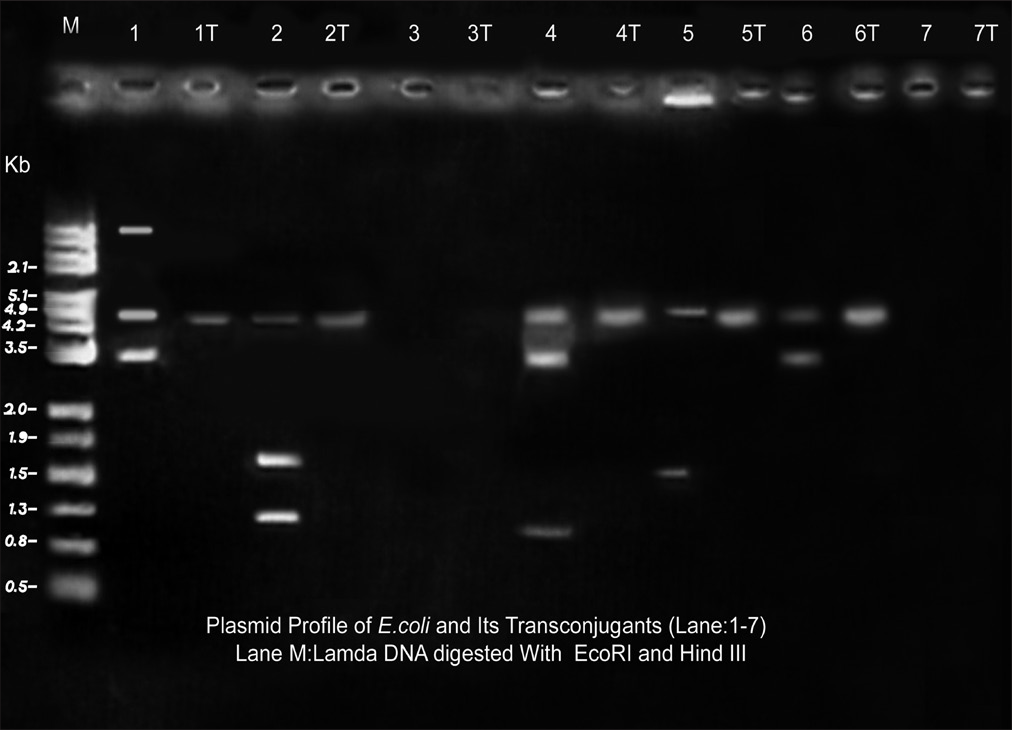
Plasmid profiles of *E.coli* isolates and its transconjugants given in lane 1-14, M: Marker Lambda DNA digested with EcoRI and HIND III

Conjugative plasmid which mediates multiple resistances was demonstrated by conjugation experiment. The results of transconjugation show that all the transconjugant colonies were resistant to ampicillin, imipenem and cotrimoxazole [Table 2]. All the transconjugants were carrying 4.8 kb plasmid that may be co-transferred either singly or in combination with other plasmids from the parents [Figure 1]. However, conjugation analysis revealed that not all plasmids were transferable by conjugation. Conjugation studies suggest that 4.8-kb plasmid contributes in resistance to ampicillin, imipenem and cotrimoxazole. No attempt was made in this study to determine the homogeneity of 4.8-kb plasmid by compatibility grouping or restriction analysis.

**Table 2:** Characteristic features of some representative strains of *E. coli* donors and transconjugants

Bacterial strain no.	Resistance pattern	Plasmid size (Kb)	Transconjugants resistance pattern	Plasmid size (Kb)
*E.coli* 1	Ap. I, Co, Na, Nor	25, 4.8, 2.6	Ap. I, Co	4.8
*E.coli* 2	Ap. I, Co, Nor, Cip, Ca	4.8, 1.6, 1.2	Ap, I, Co	4.8
*E.coli* 3	Ap. I, Co, Na, Cip, Ce	4.8, 3.2	Ap. I, Co	4.8, 3.2
*E.coli* 4	Ap. I, Co, Chl	-	-	-
*E.coli* 5	Ap. I, Co, Chl, Na	4.8, 1.5	Ap. I, Co	4.8
*E.coli* 6	Ap. I, Co, Na, Nor, Cip,	4.8, 2.0, 1.7	Ap. I, Co	4.8, 1.7
*E.coli* 7	Ap, I, Co, Na, Ca, Ci	4.8, 1.5, 1.2	-	-
*E.coli* 8	Ap. I, , Co, Nor, Na	4.8, 1.4	Ap, I, Co	4.8, 1.4
*E.coli* 9	Ap. I, Co, Chl, Na, Cip, Ca, Ci	25, 4.8, 2.8	Ap, I, Co	4.8
*E.coli* 10	Ap, I, Co, Na, Nor, Cip	4.8, 1.2	Ap, I, co,	4.8

Ap: Ampicillin, I: Imipenem, Co: Cotrimoxazole, Chl: Chloramphenicol, Cip: Ciprofloxacin, Na: Nalidixic acid, Nor: Norfloxacin, Ca: Cefotaxime, Ce: ceftazidime, Ci: Ceftriaxone

## CONCLUSION

It has been shown that there is an increase in prevalence of drug resistance among *E. coli* isolates in our region, and majority of the antibiotic resistances were due to the acquisition of plasmid-carrying antibiotic-resistance genes. Conjugal transfer of plasmids has greatly contributed to the rapid spread of antibiotic resistance among *E. coli* isolates. Restriction of use of antibiotics may play a role in decreasing the emergence of resistant bacterial strains.
